# Synthesis of Carbon Nanodots from Sugarcane Syrup, and Their Incorporation into a Hydrogel-Based Composite to Fabricate Innovative Fluorescent Microstructured Polymer Optical Fibers

**DOI:** 10.3390/gels8090553

**Published:** 2022-09-01

**Authors:** Gabriel Perli, Marco C. P. Soares, Thiago D. Cabral, Diego L. Bertuzzi, Julio R. Bartoli, Sébastien Livi, Jannick Duchet-Rumeau, Cristiano M. B. Cordeiro, Eric Fujiwara, Catia Ornelas

**Affiliations:** 1 Institute of Chemistry, University of Campinas, Campinas 13083-970, Brazil; 2 Ingénierie des Matériaux Polymères, Université de Lyon, CNRS, UMR 5223, INSA Lyon, F69621 Villeurbanne, France; 3 Laboratory of Photonic Materials and Devices, School of Mechanical Engineering, University of Campinas, Campinas 13083-860, Brazil; 4 “Gleb Wataghin” Institute of Physics, University of Campinas, Campinas 13083-859, Brazil; 5 School of Chemical Engineering, University of Campinas, Campinas 13083-852, Brazil

**Keywords:** fluorescent hydrogels, sugarcane-based carbon dots, efficient fluorescence, advanced materials

## Abstract

Carbon nanodots (CNDs) are interesting materials due to their intrinsic fluorescence, electron-transfer properties, and low toxicity. Here, we report a sustainable, cheap, and scalable methodology to obtain CNDs from sugarcane syrup using a domestic microwave oven. The CNDs were characterized by infrared spectroscopy, dynamic light scattering, atomic force microscopy, absorption, and emission spectroscopies. The CNDs have 3 nm in diameter with low polydispersity and are fluorescent. A fluorescent hydrogel–CNDs composite was obtained using gelatin polypeptide as the polymeric matrix. The new hydrogel–CNDs composite was incorporated in the cavities of a double-clad optical fiber using an innovative approach that resulted in a microstructured polymer optical fiber with intrinsic fluorescence. This work shows a promising alternative for the fabrication of fluorescent materials since the CNDs synthesis is sustainable and environmentally friendly. These CNDs might substitute the rare-earth and other heavy metals of high cost and toxicity, which are usually incorporated in double-clad fibers for applications on lasers, amplifiers, and spectroscopy.

## 1. Introduction

Carbon Nanodots (CNDs) are nanoparticles constituted by a carbon core and superficial functional groups, notable for their quasi-spherical and discrete morphologies, usually with dimensions lower than 10 nm [1,2,3,4]. CNDs present unique assets such as strong fluorescence both in solution and in the solid state, high photostability, low toxicity, high biocompatibility, high solubility in water, chemical robustness, and are easily doped and chemically modified [5,6,7,8,9]. In terms of charge transfer chemistry, CNDs have the particularity of acting as either electron donors or acceptors [10]. These properties are intrinsic to CNDs and distinct from other carbon-based nanostructures such as graphene, fullerene, and carbon nanotubes.

CNDs were discovered by Scrivens and co-workers as a fluorescent by-product of single-walled carbon nanotube synthesis [11]. Since then, several methodologies were developed to obtain CNDs that involve top-down nanocutting procedures and bottom-up organic approaches, which include arc discharge, laser ablation, electrochemical oxidation, combustion methods, hydrothermal carbonization, templated carbonization, pyrolysis in concentrated acid, and microwave irradiation [1,4,12,13]. Some of these processes are difficult to control, resulting in CNDs with high polydispersity. The size, polydispersity, and surface functional groups nature depend on the synthetic methodology used to obtain the CNDs [1].

There is a continuous search for greener and cheaper methods to produce CNDs with low polydispersity from renewable sources [14,15,16,17,18]. Nature offers a plethora of carbon sources that has motivated the investigation of a wide variety of carbon sources as raw materials to obtain CNDs, which include lignin biomass, waste paper, kitchen waste, starch, orange peels, potato, grass, soy milk, papaya, eggs, coffee grounds, hair, cow manure, among others [19,20,21,22,23]. Since CNDs obtained from different sources were synthesized through diverse protocols, they present different properties with size range varying from 1 nm to 50 nm [22]. Therefore, there is room for improvement in terms of searching for reliable and renewable carbon sources and for the development of greener, cheaper, and environmentally friendly synthetic protocols.

The molecular structure of the CNDs has not been fully elucidated, but IR and Raman studies suggest that these carbon nanoparticles are not perfect crystals and that they present a high degree of structural defects and peripheral functional groups [10]. The structural defects on the carbon core and the peripheral functional groups, which are both dependent on the synthetic protocol, have a strong influence on the photoluminescence behavior of the CNDs.

The mechanism involved in the CNDs fluorescence is still under debate in the literature, and some theories propose: (i) generation and recombination of electron-hole pairs; (ii) quantum confinement effect; and (iii) defective state emission [1,24]. CNDs usually exhibit a strong absorption band in the UV region around 230 nm that may be assigned to a π → π* transition concerning sp^2^ carbons on the carbon core, and a tail extending to the visible region that might be attributed to a n → π* transition involving the peripheral functional groups that bear electron lone pairs [25,26]. Interestingly, CNDs exhibit excitation-wavelength dependent emission, and the emission wavelength is strongly affected by the electronic bandgap transitions of conjugated π-systems, surface defects, and element doping [27,28,29]. It has been demonstrated that variation of the CNDs size shifts the maximum emission wavelength, showing that maximum emissions on UV (around 350 nm), on visible (400–700 nm), and NIR (about 800 nm) were all observed depending on the CNDs carbon core size (smaller particles emit at lower wavelengths and larger particles emit at higher wavelengths) [27,30].

The unique emission properties of CNDs allied to its easily tailored nanosize and to the high abundance of carbon sources have made CNDs attractive for a wide variety of potential applications including bioimaging, drug-delivery, sensors, optoelectronics, photovoltaic devices, photocatalysis, renewable energy devices, and anti-counterfeiting materials [9,20,22,31,32,33,34,35,36,37,38]. Moreover, the semiconductor-like properties of CNDs enabled their incorporation into optical fibers to obtain optical devices with innovative features [39,40].

For instance, optical fibers containing CNDs have demonstrated interesting properties as sensors to detect low concentrations of heavy metals, based on the CNDs fluorescence quenching [39,40], or on refractive index modulations [41]. Gonçalves et al. immobilized CNDs functionalized with PEG200 and N-acetyl-L-cysteine in an optical fiber to build a sensor that detects the submicron molar concentration of Hg(II) in aqueous solution [39]. Although the optical properties of CNDs have been explored to create devices with optical fibers for sensing applications, opportunities remain for the development of other types of optical devices based on the combination of CNDs and optical fibers.

In this work, we report a new simple microwave-based methodology to obtain fluorescent CNDs with low polydispersity from sugarcane syrup, a renewable and low-cost source. The CNDs synthesis is performed by irradiation in a domestic microwave oven, and the raw material naturally contains inorganic ions that catalyze the carbonization process. The CNDs were characterized by infrared spectroscopy (IR), absorption and emission spectroscopies, dynamic light scattering (DLS), and atomic force microscopy (AFM). The fluorescent CNDs were further immobilized into a hydrogel matrix (gelatin), in order to produce a green-fluorescent polymeric composite. An innovative microstructured polymer optical fiber (mPOF) with intrinsic fluorescence was obtained by incorporating the hydrogel–CNDs composite into a double-clad optical fiber through a newly developed design.

## 2. Results and Discussion

### 2.1. Carbon Nanodots (CNDs) Synthesis and Characterization

**Figure 1:**
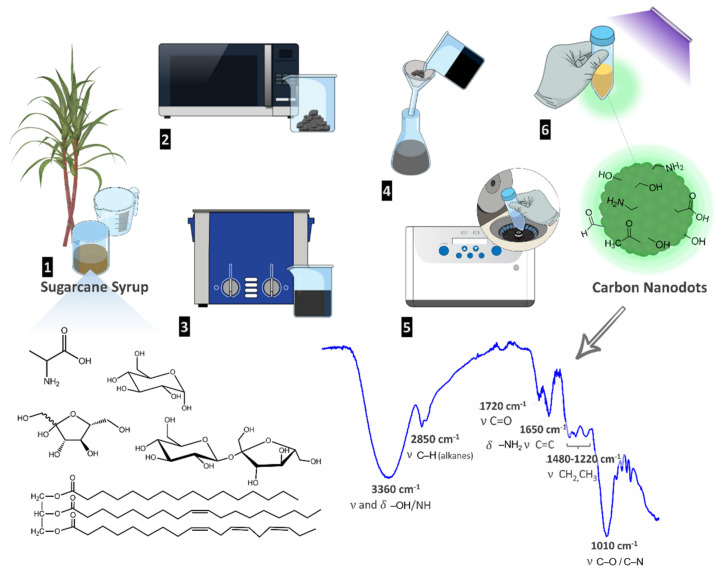
Schematic representation of the new methodology developed to obtain CNDs from sugarcane syrup; chemical structures of the major components of sugarcane syrup, schematic representation of the resulting CNDs, and FTIR spectrum of the CNDs with the assignment of the most important bands.

Due to the outstanding physical and chemical properties of CNDs, there is a continuous search for the development of synthetic methodologies to obtain CNDs from renewable sources using low-cost, fast, sustainable, low energy consumption, and scalable processes. We have developed a green, sustainable, and simple methodology to obtain CNDs from sugarcane syrup (Figure 1).

**Figure 2:**
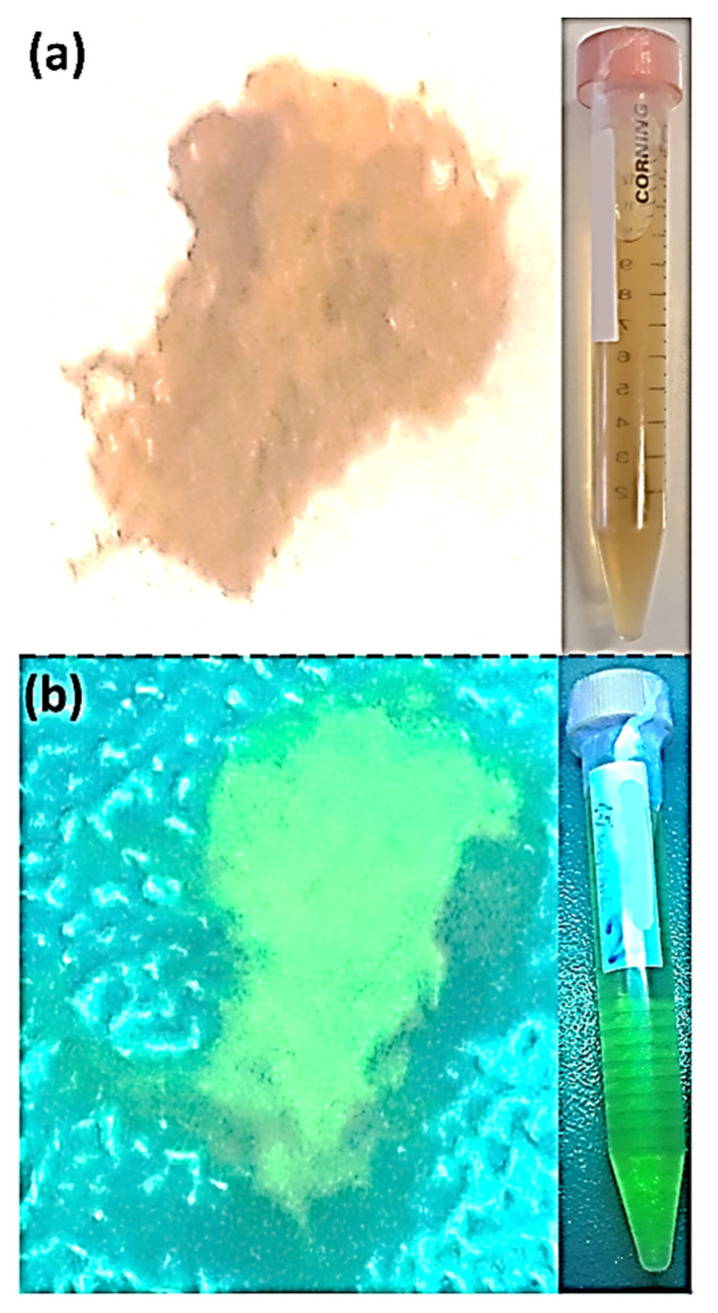
(**a**) Pictures of the obtained pure CNDs as a solid (**left**) and in aqueous solution (**right**, falcon tube). (**b**) Pictures of the obtained pure CNDs as a solid (**left**) and in aqueous solution (**right**, falcon tube) under UV light (254 nm) adapted from IEEE publisher [43].

The major components of sugarcane syrup are sucrose, glucose, fructose, amino acids, lipids, and inorganic salts containing K, Ca, Na, Fe, and Mg, among others [42]. Sugarcane syrup was diluted in deionized water and heated in a domestic microwave oven (700 W, 20 L), using a two-cycle process (1 min + 30 s) cycle. The obtained carbonized material was re-dispersed in deionized water using an ultrasound bath, and the mixture was filtered through filter paper to remove the bulk material, yielding a homogeneous brownish solution. The solution was centrifuged in tube filters with molecular weight cut-off (MWCO) of 3000 Da under 7000 g for 15 min. A yellowish suspension of CNDs with nanoparticle concentration of 32.3 mg/mL was obtained (Figure 1). FTIR spectroscopy was used to detect and identify the functional groups present in the CNDs surface (Figure 1). Typical absorption bands for alcohol, carboxylic acids, amines, aldehydes, and ketones were observed. The intense and broad-band a 3360 cm^−1^ corresponds to the stretching and bending vibrations of O–H and N–H bonds present on amines, alcohols, and carboxylic acids. The double band at 2850 cm^−1^ is assigned to C–H stretching on alkanes. The peak at 1720 cm^−1^ corresponds to the C=O bending on carboxylic acids, aldehydes, and ketones. The presence of amines is evidenced by the bands at 1650 cm^−1^ (N–H bending) and at 1040 cm^−1^ (C–N stretching vibrations). The presence of these functional groups bearing lone electron pairs contribute to the CNDs fluorescence and make these nanoparticles polar and water-soluble. Moreover, these functional groups on the CNDs periphery allow their further functionalization through covalent attachment or interaction with other nanomaterials through hydrogen bonding. The resulting CNDs are fluorescent in solution and in the solid-state after lyophilization, which was easily confirmed using a UV lamp with a wavelength of 254 nm (photos of CNDs in solution and powder in Figure 2 [43]; for absorption and emission spectra, see section photophysical properties of CNDs and hydrogel-CNDs composite).

**Figure 3:**
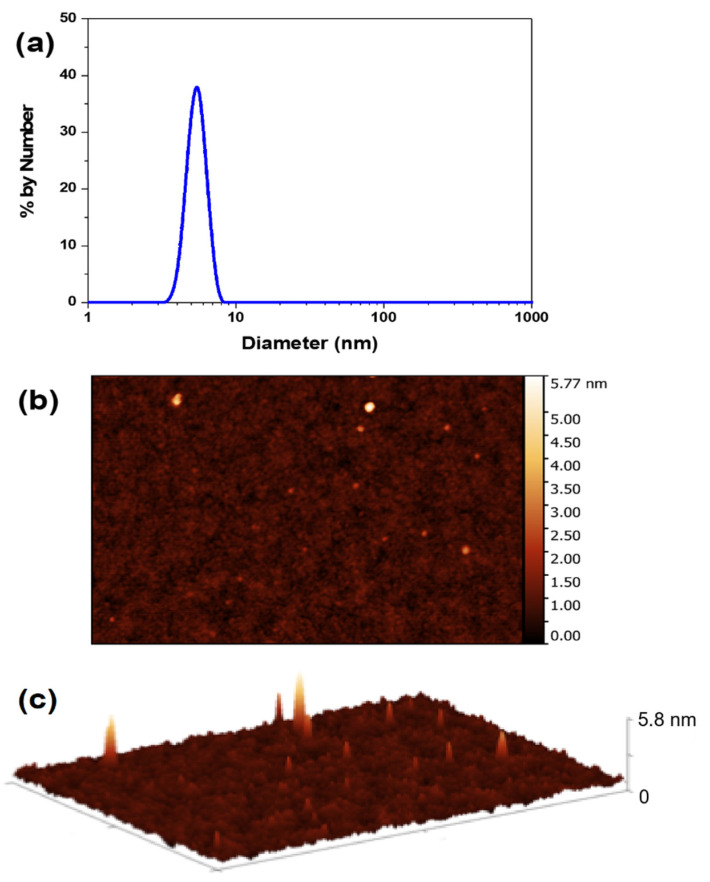
(**a**) DLS data obtained for CNDs in aqueous solution. (**b**) 2D AFM height image of CNDs deposited on a mica surface. (**c**) 3D topographic AFM image of CNDs deposited on a mica surface.

Particle size distribution was assessed through DLS analysis (Figure 3a), which reveals particles with an average hydrodynamic diameter of 5 nm, with low polydispersity. It is important to note that there is no evidence for the presence of other populations of particles with higher diameters, demonstrating the efficacy of our newly developed synthetic protocol.

The size of the CNDs was also analyzed in the solid-state by AFM using a Park NX10 Atomic Force Microscope, with an out-of-plane resolution of 0.006 nm. The sample was prepared by adding a drop of CNDs aqueous solution into a freshly cleaved mica surface. Figure 3b,c show the 2D AFM height image and the 3D topographic AFM image of the CNDs on the mica surface. In this AFM equipment, spherical nanoparticles result in circular bidimensional profiles observed on the image’s plane. Therefore, it is possible to infer that the heights indicated by the color scale of the AFM image correspond to the diameters of the individual particles. The AFM images reveal the presence of semispherical nanoparticles with average dimensions of 3.0 nm (sizes vary between 2.0 nm and 5.8 nm).

When comparing the values obtained by DLS and AFM these values are consistent because the size obtained by DLS refers to the nanoparticle’s hydrodynamic diameter in solution that includes at least one layer of surrounding solvent, whereas the AFM images are acquired in the solid state on a flat surface in which a slight flattening of the nanoparticle is expected due to interaction with the mica surface and contact with the AFM probe [44,45]. Moreover, the 3.0 nm size is consistent with CNDs with green fluorescence emission, according to previous reports [30].

### 2.2. Preparation of Hydrogel–CNDs Fluorescent Composite

In order to utilize the CNDs in the fabrication of fluorescent optical fibers, the CNDs were first immobilized in a hydrogel matrix using gelatin as the polymeric gelator. Gelatin is a natural high molecular weight polypeptide biopolymer derived from the hydrolysis of collagen. Gelatin easily forms thermoreversible hydrogels in aqueous solutions in concentrations as low as 1% *w*/*w*, by forming a micro-structural network [46]. Gelatin possesses both positively and negatively charged amino acids being a unique polyampholyte polypeptide, which confers an excellent versatility to this hydrogel material. Moreover, gelatin presents a series of advantages when compared to the more common acrylic and glycolic based hydrogels such as low-cost; wide commercial availability; low viscosity before curing; and spontaneous heat-induced curing; and non-aggressive crosslinking conditions such as photocure equipment, extreme pH, radical initiators, or metal-based catalysts [47,48]. Gelatin hydrogels have been successfully used in fiber optic devices for sensing applications [49]. A previous report showed that a membrane formed by a gelatin-CNDs composite performed as a fluorescence sensor for the detection of potassium [50].

The preparation of the hydrogel matrix started by dissolving 3 g of colorless gelatin in 94 mL of deionized water. The solution was heated at 90 °C and kept under magnetic stirring to eliminate the dissolved air bubbles. The presence of air bubbles is a common problem in hydrogel matrixes, which has a negative impact on the optical properties of the final materials [51,52]. To the transparent gelatin solution, 6 mL of the CNDs solution (32.3 mg/mL) were added and stirred until complete homogenization. The resulting yellowish solution was cooled in the refrigerator at 10 °C overnight. A thermoreversible hydrogel was obtained through the formation of a micro-structural tridimensional network [53]. Above 40 °C, the aqueous solution of gelatin behaves as an homogeneous solution of macromolecules with a typical molar mass of 2 × 105 Da, that assume random coil configurations (single polypeptide chains, termed α-chains) that may be entangled [54]. When these α-chains are cooled further, they undergo a coil-to-helix transition that leads to the formation of a tridimensional network with water molecules and the consequent gelation process [55]. During gelation, the CNDs remained within the hydrogel matrix, probably stabilized by hydrogen bonding between the terminal functional groups on the CNDs surface and the amino acids present on the gelatin polymer. The resulting hydrogel-CNDs composite presents a yellowish coloration that fluoresces upon irradiation with UV light (254 nm).

The refractive index (RI) of the hydrogel–CNDs composite was evaluated with a MISCO PA 202 Refractometer before and after curing, presenting the values of 1.3389 and 1.3390, respectively. Such values are close to the one measured for DI water (1.3330) and lower than that of PMMA (1.49) [56], indicating that the total internal reflection in the PMMA–hydrogel interface will prevent the fluorescence from leaking back into the hydrogel-filled holes.

### 2.3. Photophysical Properties of CNDs and Hydrogel-CNDs Composite

**Figure 4:**
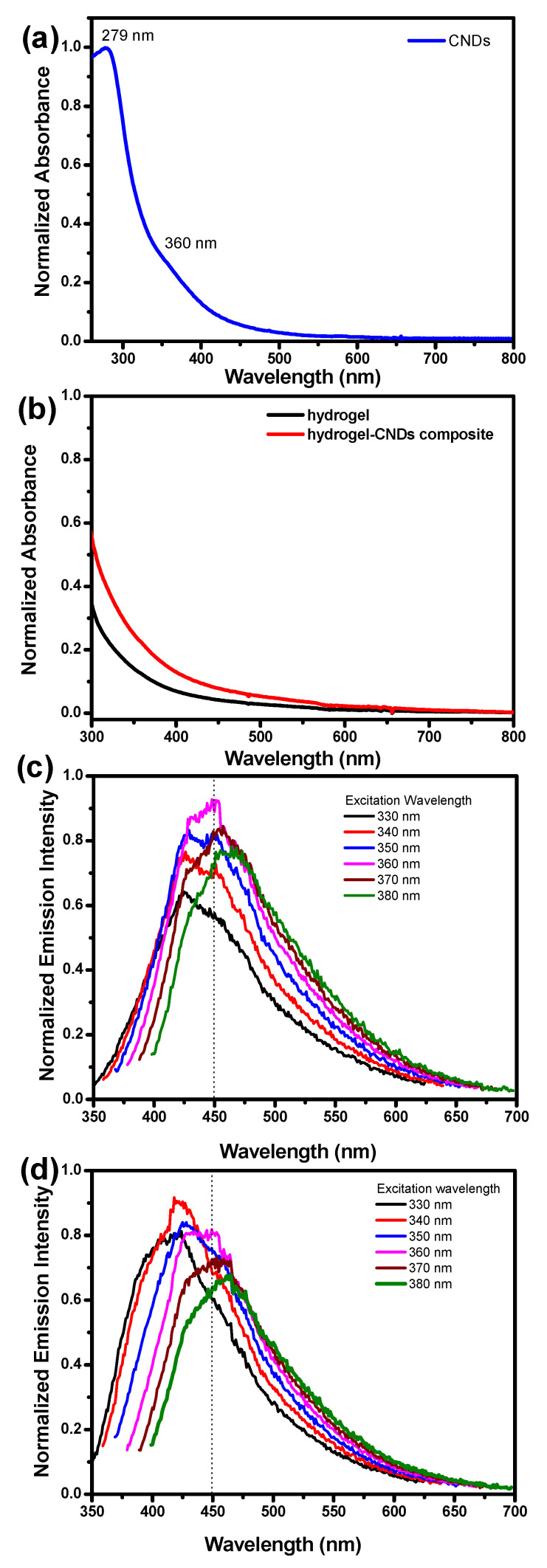
(**a**) UV-Vis absorbance spectrum of the CNDs in aqueous solution; (**b**) UV-Vis absorbance spectra of hydrogel and hydrogel–CNDs composite; (**c**) emission spectra of the CNDs in aqueous solution; (**d**) emission spectra of the hydrogel–CNDs composite.

Absorbance spectra for CNDs, hydrogel matrix, and hydrogel–CNDs composite were measured and are presented in Figure 4a,b. The CNDs present an absorption band with λ_max_ at 279 nm that corresponds to the π → π* transition assigned the sp^2^ carbons from the CNDs core, which is within the expected range for CNDs with 3–5 nm (Figure 4a). The CNDs also present a shoulder at 360 nm with a tail extending to the visible range assigned to the n → π* transitions involving the electron lone pairs from functional groups present at the CNDs periphery.

The absorption spectrum of the hydrogel matrix confirms the high transparency of this material in the visible range (Figure 4b). In contrast, the absorption spectrum of the hydrogel–CNDs composite shows a higher absorption in the 360 nm region with a tail in the visible region related to the absorption spectra of the CNDs encapsulated in the hydrogel matrix (Figure 4b).

The emission spectra of CNDs in aqueous solution and in the hydrogel–CNDs composite are dependent on the excitation wavelength, which is a well-known characteristic of CNDs (Figure 4c,d). The emission spectrum of the CNDs in solution shows that the maximum intensity of the fluorescence emission is around 450 nm, obtained when the solution is irradiated with an excitation wavelength of 360 nm. In contrast, the emission spectrum of the hydrogel–CNDs composite shows that the maximum intensity of the fluorescence emission is around 420 nm, obtained when the solution is irradiated with an excitation wavelength of 340 nm. Indeed, the emission spectra of the CNDs were blue-shifted upon encapsulation in the hydrogel matrix when comparing to the CNDs in solution. This blue-shift might be explained by the changes in the microenvironment around the CNDs. Emissions at shorter wavelengths are usually observed when the surrounding environment destabilizes the excited state of the fluorophore groups on the nanoparticle’s surface. Therefore, our data suggest that upon immobilization inside the hydrogel tridimensional network, new intermolecular interactions arise between the functional groups in the periphery of the CNDs and amino acid residues in the polypeptide causing the blue-shift on the emission spectra [57].

### 2.4. Fabrication of Microstructured Polymer Optical Fibers (mPOF) Using the Hydrogel–CNDs Composite

In this work, we used the hydrogel–CNDs composite to fabricate a microstructured polymer optical fiber (mPOF) with intrinsic fluorescence. The mPOFs consist of waveguides containing well-defined hole patterns in their cross-sections, allowing the control of the light-guiding through the appropriate design and configuration of the holes [58]. The holes can be filled with different materials and substances, providing direct interaction between the enclosed materials and the evanescent field of the light throughout the whole fiber length [59]. Among the different possible designs for mPOFs, the double-clad fiber is one that patterns the holes to create an internal clad (usually filled with air) for improving the guiding efficiency [60].

The double-clad design has been successfully employed in several applications, including the fabrication of fiber lasers with enhanced Q-switching and high-efficiency amplifiers. For these applications, the fibers are usually doped with rare-earth elements [61] such as Nd [62], Yb [60,63,64,65,66,67,68], Er [69,70,71], Tm [72,73], and combinations of Er-Yb [74,75] to explore the fluorescence of those chemicals. Moreover, given the high efficiency to confine light, the double-clad design also finds applications in fluorescence spectroscopy and biomedicine, where it can be used for endoscopy, intravascular imaging, and optical coherence tomography [76,77,78,79]. Despite their inherent versatility, these fibers are commonly combined with rare-earths and other heavy metals with superior fluorescence, like inorganic quantum dots (QDs), of high cost and toxicity. An alternative to this cost and toxicity problem is to substitute such materials for the harmless CNDs, by proper immobilizing the nanoparticles into the fiber holes. For this purpose, here we used the hydrogel–CNDs composite, which is a green, sustainable, safe, and biodegradable composite obtained from biomass with low cost.

**Figure 5:**
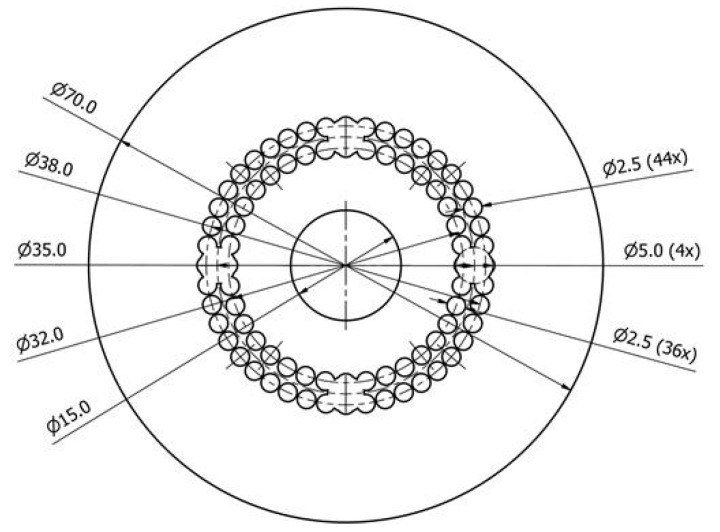
Newly developed design of double-clad optical fiber.

Here, we report an innovative design of waveguide, which is essentially different from the ones of other double-clad mPOFs previous fabricated. In other works, the reported cavities are arranged according to hexagonal profiles [60,65,80]; the fibers create a pattern of tangential circular cavities for field enhancement [81]; or the fibers present successive layers of non-tangential cavities, both in hexagonal and circular patterns [82]. The design developed in this work presents advantages for the liquid matrix incorporation. The presence of the central cavity of higher diameter and of the two tangential layers of circular cavities, with four diametral opposed holes of higher diameters, facilitates the introduction of the hydrogel containing CNDs by both external pressure and capillary forces (Figure 5). That is due to the wide and symmetrical distribution of hollow spaces through the cylindrical volume.

**Figure 6:**
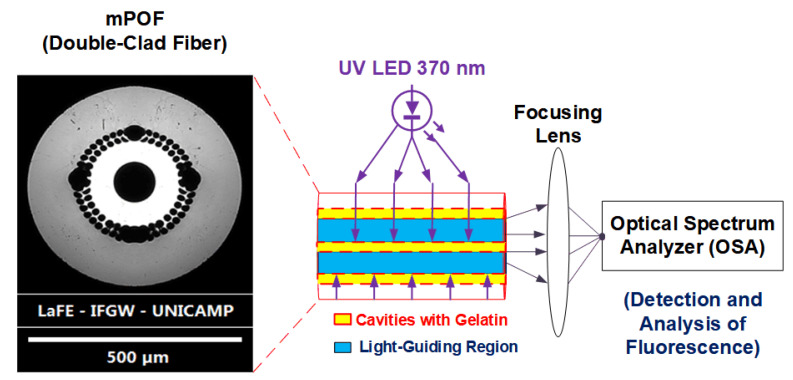
Setup for evaluation of the mPOF fluorescence and a micrograph of the fiber’s cross section.

The mPOF fiber was fabricated by pulling a structured PMMA preform on a POF draw tower in a two-step process. The preform started as a PMMA rod with an external diameter of 70 mm. Using a CNC drilling machine, several holes were introduced in the preform, prior to the drawing process. On the first step of the drawing process, the diameter of the preform is reduced from 70 mm to 12 mm, while the second drawing step reduces the 12 mm-preform to a fiber with external diameter of ~500 μm. Figure 6 shows the cross-section of the final fiber obtained.

The non-cured solution of the hydrogel–CNDs composite was introduced into the holes of the mPOF by using a syringe attached to one end of the fiber. The two ends of the mPOF were sealed, and the system was left on the refrigerator overnight, allowing the thermal crosslinking of the hydrogel matrix and the immobilization of the composite.

Light is directed to the fiber in a direction normal to its circumference, allowing it to get transmitted through the holey structure and to excite the fluorescent material inside the central hole. Due to the contrast of refractive indexes (RIs), the fluorescence emitted by the CNDs is confined and guided through the inner PMMA ring (RI ≈ 1.49) [56] and can be captured by a photodetector or spectrometer positioned by the tip of the fiber. Therefore, a (370 ± 10) nm LED operated at 48 mA was positioned perpendicularly to the axis of an mPOF piece, and it was used to externally irradiate the fiber. The visible light emerging from the tip is finally focused by an external lens to an HR4000 Spectrometer (Ocean Optics, Largo, FL, USA) for detection and analysis. This full setup and a micrograph of the mPOF cross section are shown in Figure 6.

Due to the absorption properties of CNDs and the immobilization of the composite into the mPOF cavities, the mPOF is not expected to lose power due to the intrinsic mechanisms of molecular absorption. On the other hand, this factor does not prevent the light guided through the fiber (including both the UV excitation and the fluorescent emission) to lose intensity due to attenuation or scattering by the macromolecules or by the nanoparticles themselves.

The application of the hydrogel–CNDs composite for obtaining a microstructured optical fiber with intrinsic fluorescence was verified by analyzing the light guided by the new mPOF system. Once the internal volume of the cavities of the mPOF is very low (diameter on the order of micrometers), the total number of luminescent emitters inside it is very reduced. Thus, a relatively low total intensity was expected to be verified on the fiber tip, requiring the experiment to be performed in the absence of environmental light.

**Figure 7:**
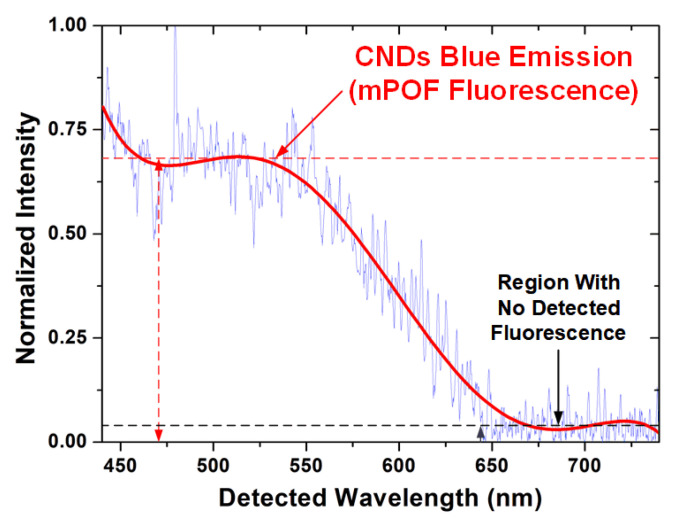
Fluorescence emitted by the mPOF with the composite of gelatin–CNDs.

The spectral data collected in the visible emission of interest collected from the mPOF are shown in Figure 7. It is possible to notice on Figure 7 that the fluorescence with the highest intensity is on the range 445–550 nm, as expected for the hydrogel–CNDs composite based on its emission spectra. Above this range, the fluorescence is progressively lower, and no emission is observed above 650 nm. Especially for the maximum intensity range, there is a substantial noise (e.g., at 470 and 475 nm) impairing the signal-to-noise ratio (SNR). It is probably a consequence of the low number of fluorescent particles present in the microchannels, which reduces the detected intensity as predicted.

The detection of the fluorescence on the range predicted by Figure 6 shows that the immobilization into the fiber does not shift the emission and that the methodology of retaining the CNDs with hydrogel into the microstructured fiber is effective for the fabrication of a fluorescent mPOF.

The fluorescent emission and the SNR could then be further improved using a higher-powered LED, by increasing the concentration of CNDs in the hydrogel–CNDs composite, or by increasing the diameter of the central mPOF hole (enhancement of the number of emitters).

## 3. Conclusions

Here we reported a sustainable, cheap, scalable, and straightforward methodology to obtain carbon nanodots with low dispersity. The resulting CNDs have 3 nm in average and are fluorescent upon irradiation with UV light. A fluorescent hydrogel–CNDs composite was obtained using cheap gelatin polypeptide as the polymeric matrix. The CNDs emission spectrum blue-shifted upon incorporation in the hydrogel tridimensional network. Remarkably, the stabilization of the CNDTs in the polymeric matrix did not significantly affect the quantum yield, reducing from 34.5% to 33.1%. The new hydrogel–CNDs composite was incorporated into the cavities of a double-clad optical fiber fabricated with an innovative design. The hydrogel–CNDs composite was introduced into a double-clad mPOF where the central empty hole is surrounded by a PMMA ring, which is, in turn, limited by two tangential layers of air-filled holes, forming an external ring with lower refractive index. The fluorescent fiber showed similar emission profiles than the composite when UV-irradiated. Therefore, this work demonstrates a potential alternative for the substitution of rare-earths and other inorganic heavy metals (which present high costs and toxicity) for applications in microstructured optical fibers.

## 4. Experimental Section

### 4.1. Materials and Methods

Sugar cane syrup was obtained from a local plant (Usina da Pedra, SP, Brazil), in commercial grade. Contrary to molasses that is usually obtained as a residue from the ethanol production, the syrup is produced as a step from the sugar process, by evaporating part of the clarified broth to concentrate the sucrose without crystallizing it.

### 4.2. Synthesis of CNDs from Sugar Cane Syrup

The sugarcane syrup (5 mL) was diluted with deionized water (5 mL) and placed into a ceramic crucible. The mixture was heated in a domestic microwave oven (700 W, 20 L) through two heating cycles: (i) 1 min followed by (ii) a second cycle of 30 s. After the first heating cycle, the microwave oven was carefully opened to relieve the internal pressure. After the carbonizing process, the ceramic crucible was left to cool up to room temperature. The carbonized solid was scraped out and redispersed in deionized water (30 mL) using an ultrasound bath for 1 h. The ultrasonicating process was repeated until the fully consumption of the carbonized bulk material.

### 4.3. Purification of the CNDs

The mixture was initially filtered through filter paper to remove the bulk carbonized material. The filtered solution containing the CNDs was further filtered using centrifugal filter tubes with molecular weight cut-off (MWCO) of 3000 Da (Merck Millipore, Danvers, MA, USA). The ultracentrifugation process was carried out under 7000 g (7873 rpm) for 15 min. The dispersion of CNDs was washed with deionized water until the centrifugal filtered water exhibits absorbance close to zero (absorbance < 0.005 UA). The sample was lyophilized resulting in 135 mg of a dark brown solid (yield of 67.5% in wt.). Part of this solid material was subsequently redispersed in deionized water to generate a CND solution in a concentration of 32.3 mg/mL.

### 4.4. Preparation of Hydrogel–CNDs Composite

In a 250 mL beaker, 3 g of colorless and odorless gelatin (food grade) was dissolved in 94 mL of deionized water. The solution was heated at 90 °C under magnetic stirring until the elimination of the air bubbles dissolved in the solution (temperature control is crucial to avoid degradation of the polypeptide material). Then, 6 mL of the CND dispersion (32.3 mg/mL) was added to the transparent gelatinous solution. The mixture was kept under magnetic stirring for 5 min until the complete homogenization. Finally, the mixture was taken to the refrigerator and stored overnight at 10 °C. A yellowish hydrogel was obtained.

### 4.5. Preparation of Microstructured Polymer Optical Fiber (mPOF) with Hydrogel–CNDs Composite

The mPOF fiber was fabricated by pulling a structured poly(methylmethacrylate), PMMA, preform on a POF draw tower in a two-step process. The preform started as a PMMA rod with an external diameter of 70 mm. Using a CNC drilling machine, several holes were introduced in the preform. This fabricated preform has two rings of 2.5 mm holes, the innermost ring has 36 equally spaced holes, whereas the outer ring has 44 holes. A group of 4 larger holes (5 mm diameter) centered along the midpoint between the inner and outer rings are placed in the cardinal directions. The preform also contains a central hole 15 mm wide. On the first step of the drawing process, the diameter of the preform is reduced from 70 mm to 12 mm, while the second drawing step reduces the 12 mm-preform to a fiber with external diameter of ~500 μm. Drawing parameters for the first step are: furnace temperature of 190 °C, preform feed speed of 0.5 mm/min, and drawing speed around 0.02 m/min. The same furnace temperature was used for the second step, but now with 5 mm/min preform feed speed and around 3 m/min of drawing speed. The same procedure for preparing the hydrogel–CNDs composite was repeated, but, prior to curing, part of the solution was introduced into the holes of the mPOF by using a syringe attached to one end of the fiber. The two ends of the mPOF were sealed, and the system was left on the refrigerator overnight, allowing the thermal-crosslinking of the matrix and the immobilization of the hydrogel–CND composite.

### 4.6. Fourier Transform Infrared Spectroscopy (FTIR)

FTIR spectrum of the CNDs was acquired in an Antaris FTIR-NIR Absorption Spectrophotometer (Thermo Fisher Scientific, Waltham, MA, USA) in ATR reflectance mode. For the FTIR analysis, the solid CND sample was directly placed onto the ATR crystal.

### 4.7. Dynamic Light Scattering (DLS)

The CNDs’ diameter was assessed by dynamic light scattering (DLS). The measurement was carried out at 25 °C on a Zetasizer Nano-ZS ZEN3600 instrument (Malvern Instruments, Malvern, UK) equipped with a 4 mW He-Ne laser with a light wavelength of 632.8 nm, and backscattering angle of 173°, using a disposable DTS 1070 cell. The solid sample of CNDs was redispersed in deionized water at a concentration of 1 mg/mL. Data are presented as % by number.

### 4.8. Atomic Force Microscopy (AFM)

AFM images of the CNDs were obtained with a Park NX10 Atomic Force Microscope (AFM, Park Systems, Suwon, Korea), with a scan width of 100 × 100 μm, z-hub of ca. 15 μm, and out-of-plane resolution of 0.006 nm. The sample was prepared by diluting the original solution of CNDs (32.3 mg/mL) in deionized water (100× dilution), dropping it on a freshly cleaved mica surface and allowing it to dry for 24 h.

### 4.9. Absorption Spectroscopy in the UV-Vis

The UV-Visible absorption spectra were acquired using a 1 cm cuvette in an Agilent 8453 UV-Vis Spectrophotometer (Agilent Technologies, Santa Clara, CA, USA).

### 4.10. Emission Spectroscopy

The emission spectra were obtained in a Varioskan LUX Multimode Microplate Reader (Thermo Fisher Scientific, Waltham, MA, USA), by irradiating it with wavelengths ranging from 300 to 430 nm.

### 4.11. Refractive Index

The refractive indexes were evaluated using a MISCO PA 202 Refractometer (Palm Abbe, Cleveland, OH, USA). A liquid suspension is dropped on the analytical surface of the equipment, which indicates the index. In the case of the hydrogels, there may be a slight variation of this index over time due to temperature changes and to the curing process of the drop. Therefore, the value of the index was evaluated immediately after dropping the solution on the equipment, and after the reading became stationary, which usually takes from 15 min to 1 h depending on the environmental temperature. On the other hand, due to the high amount of occluded water, the indexes are very close to the one obtained for water.

### 4.12. Quantum Yield

Quantum yields for CNDTs in water and the hydrogel matrix were calculated according to Williams and co-authors (Equation (1)) [83].

(1) $$QY_{C}=\frac{QY_{R}\;F_{C}A_{R}\eta_{C}^{2}}{F_{R}A_{C}\eta_{R}^{2}}\;$$

where *QY* is the quantum yield, *F* is the area of the fluorescence spectrum at 350 nm, *A* is the absorbance for the samples at low concentration (absorbance < 0.1 AU), *η* is the index of refraction. Note that *C* denotes carbon nanodots, and *R* denotes the reference sample. The reference sample is quinine sulfate in 0.1 M sulfuric acid, which has a *QY* of 54.5% [84].

## 5. Patents

Soares: M. C. P.: Perli, G.; Bartoli, J. R.; Fujiwara, E.; Suzuki, C. K. Patent: Register Number: BR 10 2019 014881 0, Title: “Production process of fluorescent nanoparticles (carbon dots) from sugar cane syrup”. Depositing Institution: INPI—Instituto Nacional da Propriedade Industrial, Depósito: 19/07/2019.

## References

[1] Wang Y., Hu A. (2014). Carbon quantum dots: Synthesis, properties and applications. J. Mater. Chem. C.

[2] Park Y., Yoo J., Lim B., Kwon W., Rhee S.W. (2016). Improving the functionality of carbon nanodots: Doping and surface functionalization. J. Mater. Chem. A.

[3] Ðorđević L., Arcudi F., D’Urso A., Cacioppo M., Micali N., Bürgi T., Purrello R., Prato M. (2018). Design principles of chiral carbon nanodots help convey chirality from molecular to nanoscale level. Nat. Commun..

[4] Xia C., Zhu S., Feng T., Yang M., Yang B. (2019). Evolution and Synthesis of Carbon Dots: From Carbon Dots to Carbonized Polymer Dots. Adv. Sci..

[5] Sun Y.-P., Zhou B., Lin Y., Wang W., Fernando K.A.S., Pathak P., Meziani M.J., Harruff B.A., Wang X., Wang H. (2006). Quantum-Sized Carbon Dots for Bright and Colorful Photoluminescence. J. Am. Chem. Soc..

[6] Huang X., Zhang F., Zhu L., Choi K.Y., Guo N., Guo J., Tackett K., Anilkumar P., Liu G., Quan Q. (2013). Effect of Injection Routes on the Biodistribution, Clearance, and Tumor Uptake of Carbon Dots. ACS Nano.

[7] Yuan F., Li S., Fan Z., Meng X., Fan L., Yang S. (2016). Shining carbon dots: Synthesis and biomedical and optoelectronic applications. Nano Today.

[8] Tian X.-T., Yin X.-B. (2019). Carbon Dots, Unconventional Preparation Strategies, and Applications Beyond Photoluminescence. Small.

[9] Panwar N., Soehartono A.M., Chan K.K., Zeng S., Xu G., Qu J., Coquet P., Yong K.-T., Chen X. (2019). Nanocarbons for Biology and Medicine: Sensing, Imaging, and Drug Delivery. Chem. Rev..

[10] Cadranel A., Margraf J.T., Strauss V., Clark T., Guldi D.M. (2019). Carbon Nanodots for Charge-Transfer Processes. Acc. Chem. Res..

[11] Xu X., Ray R., Gu Y., Ploehn H.J., Gearheart L., Raker K., Scrivens W.A. (2004). Electrophoretic Analysis and Purification of Fluorescent Single-Walled Carbon Nanotube Fragments. J. Am. Chem. Soc..

[12] Wang X., Qu K., Xu B., Ren J., Qu X. (2011). Microwave assisted one-step green synthesis of cell-permeable multicolor photoluminescent carbon dots without surface passivation reagents. J. Mater. Chem..

[13] Arcudi F., Đorđević L., Prato M. (2016). Synthesis, Separation, and Characterization of Small and Highly Fluorescent Nitrogen-Doped Carbon NanoDots. Angew. Chem. Int. Ed..

[14] Wu S., Weng P., Tang Z., Guo B. (2016). Sustainable Carbon Nanodots with Tunable Radical Scavenging Activity for Elastomers. ACS Sustain. Chem. Eng..

[15] Duarah R., Karak N. (2017). Facile and Ultrafast Green Approach to Synthesize Biobased Luminescent Reduced Carbon Nanodot: An Efficient Photocatalyst. ACS Sustain. Chem. Eng..

[16] Devi R., Dhamodharan R. (2018). Sustainable Process for Separating Chitin and Simultaneous Synthesis of Carbon Nanodots from Shellfish Waste Using 2% Aqueous Urea Solution. ACS Sustain. Chem. Eng..

[17] Ran X., Qu Q., Li L., Zuo L., Zhang S., Gui J., Kang Y., Yang L. (2018). One-Step Synthesis of Novel Photoluminescent Nitrogen-Rich Carbon Nanodots from Allylamine for Highly Sensitive and Selective Fluorescence Detection of Trinitrophenol and Fluorescent Ink. ACS Sustain. Chem. Eng..

[18] Ravishankar K., Shelly K.M., Narayanan A., Dhamodharan R. (2019). Rapid, Solvent-Free Synthesis of Amorphous, Photoluminescent, Carbon Nanodots from Imidazole and Maleic Anhydride Solids. ACS Sustain. Chem. Eng..

[19] Zhang Z., Sun W., Wu P. (2015). Highly Photoluminescent Carbon Dots Derived from Egg White: Facile and Green Synthesis, Photoluminescence Properties, and Multiple Applications. ACS Sustain. Chem. Eng..

[20] Sharma V., Tiwari P., Mobin S.M. (2017). Sustainable carbon-dots: Recent advances in green carbon dots for sensing and bioimaging. J. Mater. Chem. B.

[21] Xu H., Xie L., Hakkarainen M. (2017). Coffee-Ground-Derived Quantum Dots for Aqueous Processable Nanoporous Graphene Membranes. ACS Sustain. Chem. Eng..

[22] Meng W., Bai X., Wang B., Liu Z., Lu S., Yang B. (2019). Biomass-Derived Carbon Dots and Their Applications. Energy Environ. Mater..

[23] Surendran P., Lakshmanan A., Vinitha G., Gopal R., Rameshkumar P. (2019). Facile preparation of high fluorescent carbon quantum dots from orange waste peels for nonlinear optical applications. Luminescence.

[24] Zhu S., Song Y., Zhao X., Shao J., Zhang J., Yang B. (2015). The photoluminescence mechanism in carbon dots (graphene quantum dots, carbon nanodots, and polymer dots): Current state and future perspective. Nano Res..

[25] Luo Z., Lu Y., Somers L.A., Johnson A.T.C. (2009). High Yield Preparation of Macroscopic Graphene Oxide Membranes. J. Am. Chem. Soc..

[26] Baker S.N., Baker G.A. (2010). Luminescent Carbon Nanodots: Emergent Nanolights. Angew. Chem. Int. Ed..

[27] Zuo P., Lu X., Sun Z., Guo Y., He H. (2016). A review on syntheses, properties, characterization and bioanalytical applications of fluorescent carbon dots. Microchim. Acta.

[28] Gan Z., Xu H., Hao Y. (2016). Mechanism for excitation-dependent photoluminescence from graphene quantum dots and other graphene oxide derivates: Consensus, debates and challenges. Nanoscale.

[29] Yan F., Sun Z., Zhang H., Sun X., Jiang Y., Bai Z. (2019). The fluorescence mechanism of carbon dots, and methods for tuning their emission color: A review. Microchim. Acta.

[30] Li H., He X., Kang Z., Huang H., Liu Y., Liu J., Lian S., Tsang C.H.A., Yang X., Lee S.-T. (2010). Water-Soluble Fluorescent Carbon Quantum Dots and Photocatalyst Design. Angew. Chem. Int. Ed..

[31] Gupta V., Chaudhary N., Srivastava R., Sharma G.D., Bhardwaj R., Chand S. (2011). Luminscent Graphene Quantum Dots for Organic Photovoltaic Devices. J. Am. Chem. Soc..

[32] Gao X., Du C., Zhuang Z., Chen W. (2016). Carbon quantum dot-based nanoprobes for metal ion detection. J. Mater. Chem. C.

[33] Tian Z., Zhang X., Li D., Zhou D., Jing P., Shen D., Qu S., Zboril R., Rogach A.L. (2017). Full-Color Inorganic Carbon Dot Phosphors for White-Light-Emitting Diodes. Adv. Optical Mater..

[34] Arcudi F., Đorđević L., Prato M. (2017). Rationally Designed Carbon Nanodots towards Pure White-Light Emission. Angew. Chem. Int. Ed..

[35] Ji T., Guo B., Liu F., Zeng Q., Yu C., Du X., Jin G., Feng T., Zhu S., Li F. (2018). Cathode and Anode Interlayers Based on Polymer Carbon Dots via Work Function Regulation for Efficient Polymer Solar Cells. Adv. Mater. Interfaces.

[36] Li H.-J., Sun X., Xue F., Ou N., Sun B.-W., Qian D.-J., Chen M., Wang D., Yang J., Wang X. (2018). Redox Induced Fluorescence On–Off Switching Based on Nitrogen Enriched Graphene Quantum Dots for Formaldehyde Detection and Bioimaging. ACS Sustain. Chem. Eng..

[37] Arcudi F., Đorđević L., Prato M. (2019). Design, Synthesis, and Functionalization Strategies of Tailored Carbon Nanodots. Acc. Chem. Res..

[38] Chen B., Xie H., Wang S., Guo Z., Hu Y., Xie H. (2019). UV light-tunable fluorescent inks and polymer hydrogel films based on carbon nanodots and lanthanide for enhancing anti-counterfeiting. Luminescence.

[39] Gonçalves H.M.R., Duarte A.J., Esteves da Silva J.C.G. (2010). Optical fiber sensor for Hg(II) based on carbon dots. Biosens. Bioelectron..

[40] Lin H., Huang J., Ding L. (2019). A Recyclable Optical Fiber Sensor Based on Fluorescent Carbon Dots for the Determination of Ferric Ion Concentrations. J. Lightwave Technol..

[41] Yap S.H.K., Chan K.K., Zhang G., Tjin S.C., Yong K.-T. (2019). Carbon Dot-functionalized Interferometric Optical Fiber Sensor for Detection of Ferric Ions in Biological Samples. ACS Appl. Mater. Interf..

[42] Borges E., Lopes M., Amorim H. (2012). Impact of sugar cane juice chemical composition on clarification and VHP sugar quality. Int. Sugar J..

[43] Soares M.C.P., Perli G., Bartoli J.R., Bertuzzi D.L., Taketa T.B., Bataglioli R.A., Suzuki C.K., Ornelas C., Fujiwara E. Fast Microwave-Assisted Synthesis of Green-Fluorescent Carbon Nanodots from Sugarcane Syrup. Proceedings of the 2019 SBFoton International Optics and Photonics Conference (SBFoton IOPC).

[44] Ornelas C., Ruiz J., Belin C., Astruc D. (2009). Giant Dendritic Molecular Electrochrome Batteries with Ferrocenyl and Pentamethylferrocenyl Termini. J. Am. Chem. Soc..

[45] Astruc D., Ornelas C., Ruiz J. (2009). Dendritic Molecular Electrochromic Batteries Based on Redox-Robust Metallocenes. Chem. Eur. J..

[46] Han Y., Lv S. (2019). Synthesis of chemically crosslinked pullulan/gelatin-based extracellular matrix-mimetic gels. Int. J. Biolog. Macromol..

[47] Burey P., Bhandari B.R., Howes T., Gidley M.J. (2008). Hydrocolloid Gel Particles: Formation, Characterization, and Application. Crit. Rev. Food Sci. Nutr..

[48] Zhou M., Guo J., Yang C. (2018). Ratiometric fluorescence sensor for Fe3+ ions detection based on quantum dot-doped hydrogel optical fiber. Sens. Actuators B Chem..

[49] Schyrr B., Boder-Pasche S., Ischer R., Smajda R., Voirin G. (2015). Fiber-optic protease sensor based on the degradation of thin gelatin films. Sens. Bio-Sens. Res..

[50] Rahimi M., Mahani M., Hassani Z. (2019). Carbon quantum dots fluorescence quenching for potassium optode construction. Luminescence.

[51] Choi M., Choi J.W., Kim S., Nizamoglu S., Hahn S.K., Yun S.H. (2013). Light-guiding hydrogels for cell-based sensing and optogenetic synthesis in vivo. Nat. Photonics.

[52] Choi M., Humar M., Kim S., Yun S.-H. (2015). Step-Index Optical Fiber Made of Biocompatible Hydrogels. Adv. Mater..

[53] Djabourov M. (1991). Gelation—A review. Polym. Inter..

[54] Bohidar H.B., Jena S.S. (1993). Kinetics of sol–gel transition in thermoreversible gelation of gelatin. J. Chem. Phys..

[55] Finer E.G., Franks F., Phillips M.C., Suggett A. (1975). Gel formation from solutions of single-chain gelatin. Biopolymers.

[56] Beadie G., Brindza M., Flynn R.A., Rosenberg A., Shirk J.S. (2015). Refractive index measurements of poly(methyl methacrylate) (PMMA) from 0.4 to 1.6 µm. Appl. Opt..

[57] Suppan P. (1990). Invited review solvatochromic shifts: The influence of the medium on the energy of electronic states. J. Photochem. Photobiol. A Chem..

[58] Eijkelenborg M.A., Large M.C.J., Argyros A., Zagari J., Manos S., Issa N.A., Bassett I., Fleming S., McPhedran R.C., Sterke C.M. (2001). Microstructured polymer optical fibre. Opt. Express.

[59] Yang X., Yu W., Liu Z., Yang J., Zhang Y., Kong D., Long Q., Yuan T., Cao J., Yuan L. (2018). Optofluidic twin-core hollow fiber interferometer for label-free sensing of the streptavidin-biotin binding. Sens. Actuators B Chem..

[60] Limpert J., Schreiber T., Nolte S., Zellmer H., Tünnermann A., Iliew R., Lederer F., Broeng J., Vienne G., Petersson A. (2003). High-power air-clad large-mode-area photonic crystal fiber laser. Opt. Express.

[61] Zenteno L. (1993). High-power double-clad fiber lasers. J. Lightwave Technol..

[62] Chen Z.J., Grudinin A.B., Porta J., Minelly J.D. (1998). Enhanced Q switching in double-clad fiber lasers. Opt. Lett..

[63] Hideur A., Chartier T., Özkul C., Sanchez F. (2000). Dynamics and stabilization of a high power side-pumped Yb-doped double-clad fiber laser. Opt. Commun..

[64] Wang Y., Po H. (2003). Dynamic Characteristics of Double-Clad Fiber Amplifiers for High-Power Pulse Amplification. J. Lightwave Technol..

[65] Limpert J., Deguil-Robin N., Manek-Hönninger I., Salin F., Röser F., Liem A., Schreiber T., Nolte S., Zellmer H., Tünnermann A. (2005). High-power rod-type photonic crystal fiber laser. Opt. Express.

[66] Ye C., Yan P., Huang L., Liu Q., Gong M. (2007). Stimulated Brillouin scattering phenomena in a nanosecond linearly polarized Yb-doped double-clad fiber amplifier. Laser Phys. Lett..

[67] Filippov V., Chamorovskii Y., Kerttula J., Golant K., Pessa M., Okhotnikov O.G. (2008). Double clad tapered fiber for high power applications. Opt. Express.

[68] Filippov V., Kerttula J., Chamorovskii Y., Golant K., Okhotnikov O.G. (2010). Highly efficient 750 W tapered double-clad ytterbium fiber laser. Opt. Express.

[69] Doya V., Legrand O., Mortessagne F. (2001). Optimized absorption in a chaotic double-clad fiber amplifier. Opt. Lett..

[70] Amrani F., Haboucha A., Salhi M., Leblond H., Komarov A., Grelu P., Sanchez F. (2009). Passively mode-locked erbium-doped double-clad fiber laser operating at the 322nd harmonic. Opt. Lett..

[71] Abedin K.S., Fini J.M., Thierry T.F., Zhu B., Yan M.F., Bansal L., Dimarcello F.V., Monberg E.M., DiGiovanni D.J. (2014). Seven-core erbium-doped double-clad fiber amplifier pumped simultaneously by side-coupled multimode fiber. Opt. Lett..

[72] Liu C., Ye C., Luo Z., Cheng H., Wu D., Zheng Y., Liu Z., Qu B. (2013). High-energy passively Q-switched 2 μm Tm3+-doped double-clad fiber laser using graphene-oxide-deposited fiber taper. Opt. Express.

[73] Luo Z., Liu C., Huang Y., Wu D., Wu J., Xu H., Cai Z., Lin Z., Sun L., Weng J. (2014). Topological-Insulator Passively Q-Switched Double-Clad Fiber Laser at 2 μm Wavelength. IEEE J. Selec. Top. Quantum Electron..

[74] Laroche M., Chardon A.M., Nilsson J., Shepherd D.P., Clarkson W.A., Girard S., Moncorgé R. (2002). Compact diode-pumped passively Q-switched tunable Er–Yb double-clad fiber laser. Opt. Lett..

[75] Sobon G., Krzempek K., Kaczmarek P., Abramski K.M., Nikodem M. (2011). 10GHz passive harmonic mode-locking in Er–Yb double-clad fiber laser. Opt. Commun..

[76] Yelin D., Bouma B.E., Yun S.H., Tearney G.J. (2004). Double-clad fiber for endoscopy. Opt. Lett..

[77] Wang L., Choi H.Y., Jung Y., Lee B.H., Kim K.-T. (2007). Optical probe based on double-clad optical fiber for fluorescence spectroscopy. Opt. Express.

[78] Lemire-Renaud S., Rivard M., Strupler M., Morneau D., Verpillat F., Daxhelet X., Godbout N., Boudoux C. (2010). Double-clad fiber coupler for endoscopy. Opt. Express.

[79] Liang S., Sun C., Saidi A., Jing J., Liu G., Li J., Zhang J., Chen Z., Narula J. (2012). Intravascular atherosclerotic imaging with combined fluorescence and optical coherence tomography probe based on a double-clad fiber combiner. J. Biomed. Opt..

[80] Beltrán-Mejía F., Cordeiro C.M.B., Andrés P., Silvestre E. (2012). Broadband dispersion compensation using inner cladding modes in photonic crystal fibers. Opt. Express.

[81] Wiederhecker G.S., Cordeiro C.M.B., Couny F., Benabid F., Maier S.A., Knight J.C., Cruz C.H.B., Fragnito H.L. (2007). Field enhancement within an optical fibre with a subwavelength air core. Nat. Photon.

[82] Zhang H., Zhang X., Li H., Deng Y., Xi L., Tang X., Zhang W. (2017). The Orbital Angular Momentum Modes Supporting Fibers Based on the Photonic Crystal Fiber Structure. Crystals.

[83] Williams A.T.R., Winfield S.A., Miller J.N. (1983). Relative fluorescence quantum yields using a computer-controlled luminescence spectrometer. Analyst.

[84] Brouwer A.M. (2011). Standards for photoluminescence quantum yield measurements in solution (IUPAC Technical Report). Pure Appl. Chem..

